# PORCN Moonlights in a Wnt-Independent Pathway That Regulates Cancer Cell Proliferation

**DOI:** 10.1371/journal.pone.0034532

**Published:** 2012-04-11

**Authors:** Tracy M. Covey, Simran Kaur, Tina Tan Ong, Kyle D. Proffitt, Yonghui Wu, Patrick Tan, David M. Virshup

**Affiliations:** 1 Cancer and Stem Cell Biology, Duke-NUS Graduate Medical School, Singapore, Singapore; 2 Department of Biochemistry, National University of Singapore, Singapore, Singapore; 3 Department of Pediatrics, Duke University, Durham, North Carolina, United States of America; National Cancer Center, Japan

## Abstract

Porcupine (PORCN) is a membrane-bound O-acyl transferase that is required for the palmitoylation of Wnt proteins, and that is essential in diverse Wnt pathways for Wnt-Wntless (WLS) binding, Wnt secretion, and Wnt signaling activity. We tested if PORCN was required for the proliferation of transformed cells. Knockdown of PORCN by multiple independent siRNAs results in a cell growth defect in a subset of epithelial cancer cell lines. The growth defect is transformation-dependent in human mammary epithelial (HMEC) cells. Additionally, inducible PORCN knockdown by two independent shRNAs markedly reduces the growth of established MDA-MB-231 cancers in orthotopic xenografts in immunodeficient mice. Unexpectedly, the proliferation defect resulting from loss of PORCN occurs in a Wnt-independent manner, as it is rescued by re-expression of catalytically inactive PORCN, and is not seen after RNAi-mediated knockdown of the Wnt carrier protein WLS, nor after treatment with the PORCN inhibitor IWP. Consistent with a role in a Wnt-independent pathway, knockdown of PORCN regulates a distinct set of genes that are not altered by other inhibitors of Wnt signaling. PORCN protein thus appears to moonlight in a novel signaling pathway that is rate-limiting for cancer cell growth and tumorigenesis independent of its enzymatic function in Wnt biosynthesis and secretion.

## Introduction

Wnts are secreted acylated glycoproteins that can act as autocrine or short-range signaling molecules and long-range morphogens. The 19 different human Wnts regulate multiple signaling pathways, and their dysregulation is implicated in diverse disorders of development, stem cell biology, proliferation and angiogenesis [1], [2]. There is thus intense interest in manipulation of the Wnt pathway though intervention at diverse points in Wnt production and the downstream signaling cascades. One strategy to achieve broad disruption of Wnt signaling pathways is to prevent Wnt secretion through inhibition of the PORCN protein.

Porcupine (PORCN) was first discovered as a Drosophila segment polarity gene necessary for the normal distribution of Wingless (Wg), the Drosophila homolog of WNT [3]. PORCN is a member of the Membrane-Bound O-Acyl Transferase (MBOAT) family and is conserved among species [4], [5]. PORCN is a multi-pass integral membrane enzyme resident in the endoplasmic reticulum and is required for the lipid modification of Wnt proteins. Acylation by PORCN appears to be absolutely essential for the proper secretion and activity of all vertebrate Wnts [5], [6], [7], [8], [9], [10], [11] and genetic deletion of PORCN is embryonic lethal [12], [13]. PORCN has no known function beyond its role in the biogenesis of Wnts, and is therefore an attractive therapeutic target in diseases with increased Wnt signaling. Indeed, several small molecule inhibitors of PORCN function have been recently reported, including Inhibitor of Wnt Production 1 and 2 (IWP-1 and IWP-2) [14]. There are two general approaches to PORCN inhibition, pharmacologic and genetic. Notably, these methods of PORCN inhibition may give different results. This can be due to differing durations and degree of inhibition but also because genetic ablation of an enzyme can unexpectedly unmask multiple independent functions for a single gene product.

Two sites in Wnt proteins can be acylated: the serine corresponding to S209 of WNT3A is palmitoleated, while the cysteine corresponding to Cys77 is palmitoylated [4], [11], [15]. Ser209 acylation is required for WNT binding to Wntless (WLS) [16], a highly conserved multipass transmembrane protein specifically involved in Wnt secretion [17], [18], [19]. WLS transports Wnts to the plasma membrane, where they are then released into the extracellular space in a pH-dependent manner. Consistent with WLS's role in Wnt secretion, cells lacking functional WLS accumulate Wg in the Golgi [17] and mice lacking WLS have lethal developmental phenotypes consistent with a key role in Wnt signaling [20]. The role of Wnt cysteine acylation is less clear [21]. Cys77 appears to be involved in signaling activity of the secreted protein, as mutation of the site results in a secreted protein with variably reduced signaling activity [15]. Palmitoylation at this site may be necessary for binding to Frizzled or other receptors.

While PORCN is clearly critical for Wnt secretion and function, it is not known if it plays additional roles distinct from its enzymatic function in the Wnt pathway. For example, PORCN could acylate additional non-Wnt substrates. Alternatively, since PORCN is an evolutionarily ancient protein present in the simplest metazoans [22], it may have co-evolved enzyme-independent, “moonlighting” functions as well. As PORCN is both critical in development and a potential therapeutic target in human disease, it is important to fully understand the role of the PORCN protein in cells. To dissect the role of PORCN in Wnt-dependent versus Wnt-independent pathways, we compared the effect of inhibiting Wnt secretion by multiple means including PORCN knockdown, WLS knockdown, and small molecule inhibition of PORCN. We find that PORCN functions in at least two independent pathways, one that controls Wnt secretion, and a second that does not require palmitoyl transferase activity but that is rate-limiting for the growth of transformed epithelial cells and regulates expression of a distinct set of genes. PORCN's Wnt-independent role in rapid proliferation has important implications for both embryonic development and cancer.

## Materials and Methods

### Ethics Statement

All experiments conducted on mice address relevant and necessary scientific questions. These were performed using recommended anesthesia and analgesics during procedures and concluded in a timely fashion to prevent unnecessary pain or burden on the mice. All experiments were performed with the approval of the NUS Institutional Animal Care and Use Committee (IACUC).

### Cells, plasmids, and reagents

All cancer cell lines were obtained from the ATCC. The STF and STF3A cells were derived from HEK 293 cells as previously described [23]. HMEC transformed (hTERT + H-Ras-V12 + p53 knockdown) and immortalized (hTERT) cells were the gift of Mathijs Voorhoeve, Duke-NUS Graduate Medical School, Singapore. Male murine embryonic stem (ES) cells with a targeted PORCN locus that places LoxP sites upstream of exon 8 and downstream of exon 10 were made by Ozgene. PORCN null gene-targeted ES cells were selected for by Davor Solter's lab (Institute for Medical Biology, Singapore). MDA-MB-231 cells expressing pTRIPZ were made using lentiviral transduction (Open Biosystems). The shRNA sequences used were **P1**: 5′- CCT GGA TAT CCT TCC ACA GCT -3′ **P2**: 5′- TAT TTA GCC AAT AAG ACA TGG T -3′, **W1**: 5′- ACC AAG AAG CTG TGC ATT GTT -3′, **W5**: 5′- TGG ACA TTG CCT TCA AGC TAA-3′. pMKIT-HA-mPORC-D (gift from Tatsuhiko Kadowaki) was cloned into the retroviral plasmid MSCV-puro (gift from Mathijs Voorhoeve). Point and siRNA-immune mutants were made using Stratagene Quikchange site directed mutagenesis. MDA-MB-231 and MCF7 cells stably expressing wildtype and mutant PORCN were generated by retroviral transduction and selection in puromycin. The small molecule PORCN inhibitors IWP-1 and IWP-2 were generously provided by Dr. Lawrence Lum. siRNAs were from Dharmacon: Control/Non-targeting (#D-001810-01-05), PORCN 7 (#J-009613-07), PORCN 8 (#J-009613-08), WLS 5 (#J-018728-05), beta-catenin 11 (#J-093415-11).

#### RT-PCR/qPCR

Total RNA was extracted from cells using Qiagen RNeasy Kit, 1 µg RNA was reverse transcribed using Bio-Rad iScript cDNA synthesis kit, and qPCR was carried out with Bio-Rad SsoFast EvaGreen Supermix.

#### Mouse Tumor Models

For transient knockdown, 100,000 MDA-MB-231 cells were transfected with the indicated siRNAs (Control or P7) and then injected into the 4^th^ position mammary fat pad of 6 week old female NOD-SCID mice. For PORCN and WLS tumor models, 500,000 MDA-MB-231 cells in 50 µl DMEM were injected orthotopically into the 4^th^ position mammary fat pad of 6 week old female BALB/c nude mice. One week post injection, the staples were removed and tumor growth was quantified by caliper measurement. To induce PORCN knockdown, drinking water was supplemented with doxycycline at 0.5 mg/mL and refreshed every other day. Upon termination of the study, the tumors were harvested, weighed, and gene expression was analyzed.

#### Proliferation Studies

Cells were plated at 70–80% confluency in 6-well plates and transfected with the specified siRNAs at 100 nM using Dharmafect 1 following the manufacturer's protocol. 24 h post transfection, cells were trypsinized and replated at a 1∶40 to 1∶80 dilution in 12-well plates. For proliferation studies with IWP, cells were plated into 12-well plates at 10% confluency. Media containing vehicle (DMSO) or IWP-1/2 was refreshed daily. The 12-well plates were collected over a 7-day period, fixed with ice-cold MeOH, and stained with crystal violet (0.5% CV in 25% MeOH). To quantify cell number, the crystal violet was solubilized in 1% sodium deoxycholate in water and absorbance was measured at 590 nm. Parallel experiments were performed and cells were trypsinized and counted with a Beckman Coulter Counter over a 7-day period.

#### Microarray

MDA-MB-231 cells (70–80% confluent) were transfected with 50 nM siRNA for 48 hours. RNA was isolated using RNeasy purification kit from Qiagen. Labeled cRNA was prepared and hybridized to Affymetrix U133_Plus_2.0 microarrays according to the manufacturer's protocols. The array data was normalized using RMA algorithm. 1482 varying genes are detected by 1 way ANOVA algorithm (False Discovery Rate (FDR)< = 0.01). Hierarchical clustering analysis identifies significantly different groups of samples from 1482 varying genes. Analysis was carried out using Partek software.

## Results

### PORCN is required for Wnt secretion

**Figure 1:**
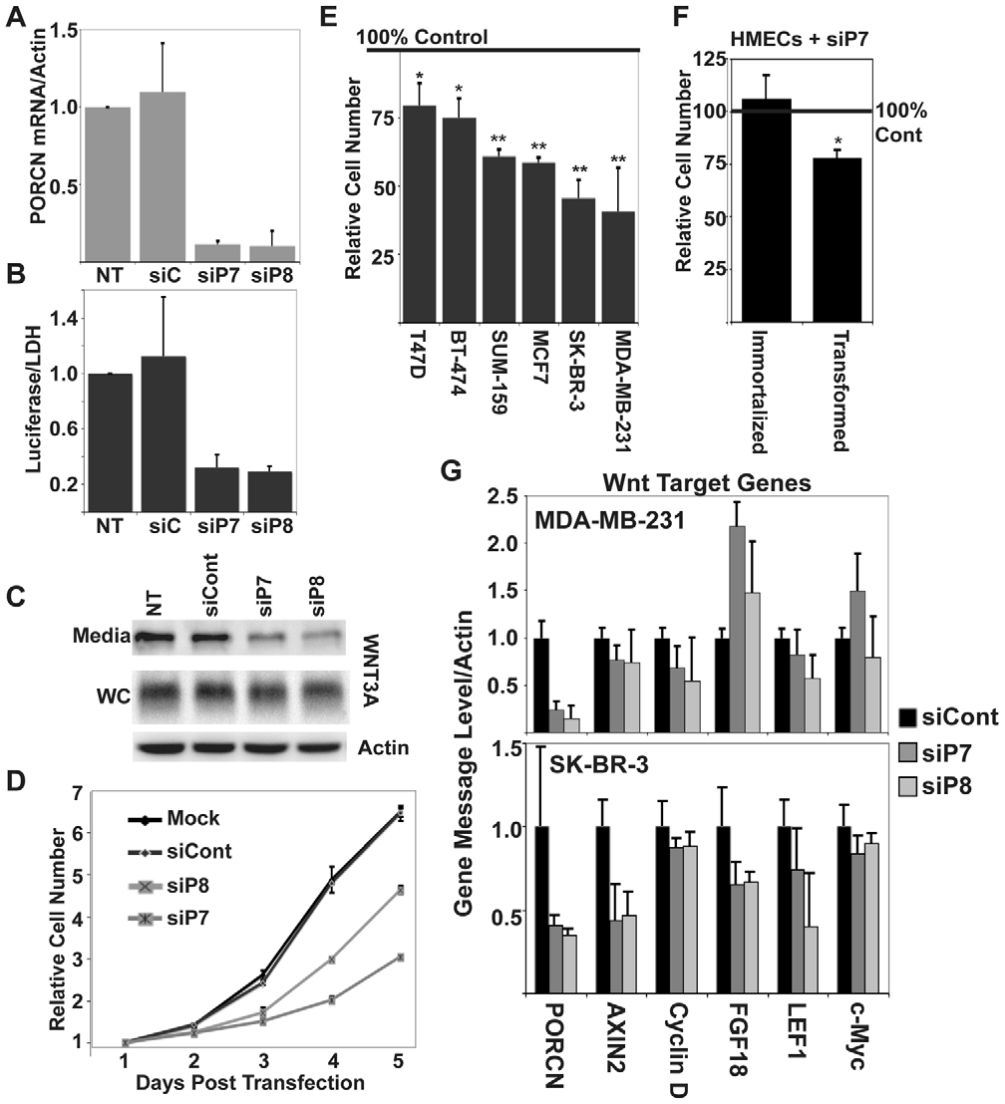
Knockdown of PORCN affects breast cancer cell proliferation. A. STF3A cells were transfected with 100 nM of the indicated siRNA and PORCN message was analyzed 48 hrs later by quantitative real time PCR. Histogram represents relative PORCN mRNA normalized to Actin mRNA. NT, not transfected; siC, siRNA control; siP7 and siP8, specific PORCN siRNAs. B. PORCN knockdown blocks Wnt/β-catenin signaling. Relative Wnt/β-catenin signaling was measured in STF3A cells transfected with 100 nM of siC, siP7, or siP8 siRNA. C. PORCN knockdown inhibits WNT3A secretion. STF3A cells were transfected with 100 nM of the indicated siRNA. Media was changed to 1% FCS media 48 hours after transfection, and collected 16 hrs later. The abundance of WNT3A was assessed in 30 µL conditioned media (Media) and 15 µg of whole cell lysates (WC) by SDS-PAGE and immunoblotting with the indicated antibodies. Actin immunoblotting demonstrates equal loading of whole cell lysates. D. Relative numbers of MDA-MB-231 breast cancer cells is decreased after PORCN knockdown. Cells were transfected with the indicated siRNAs and proliferation assessed as in Experimental Procedures. Error bars indicate standard deviation. E. PORCN knockdown slows the growth of multiple breast cancer cell lines. Cell number was assessed 4 days post-transfection of siP7 siRNA and plotted as % of proliferation of control siRNA-transfected cells. *, p<0.05; **, p<0.01 for difference from control. F. PORCN knockdown selectively slows the growth of transformed hMECs. The effect of PORCN knockdown on the growth of hMECs immortalized with hTERT or hMEC-hTERT cells transformed by expression of H-RasV12 and stable knockdown of p53 was assessed 6 days after transfection with control or siP7 siRNA. Transfection efficiency in both cell types was >80% as assessed by GFP expression. *, p<0.05 compared with control siRNA. G. PORCN knockdown alters expression of Wnt/β-catenin target genes. Relative message of PORCN and other Wnt/β-catenin target genes in MDA-MB-231 cells (top) and SK-BR-3 cells (bottom) was assessed by qRT-PCR 72-hrs after transfection with 100 nM of the indicated siRNA.

To generate tools for investigating PORCN function, we identified and validated two independent and non-overlapping siRNAs, siP7 and siP8, that target all splice variants of PORCN and gave greater than 90% knockdown of PORCN mRNA (Figure 1A) in HEK-293 cells. As expected, knockdown of PORCN in the presence of transfected WNT3A resulted in a decrease in β-catenin/TCF-driven luciferase expression in HEK-293 cells with an integrated SuperTopFlash reporter (STF cells) (Figure 1B). Likewise, knockdown of PORCN in STF cells with stable integration of WNT3A (STF3A cells) resulted in a defect of WNT3A secretion into the medium (Figure 1C), consistent with PORCN's essential role in Wnt secretion.

### Loss of PORCN inhibits breast cancer cell growth

To test if PORCN was important for breast cancer cell proliferation we first assessed its expression in a panel of breast cancer cell lines. PORCN mRNA was present in all lines tested, with MDA-MB-231 and SUM-159 cells displaying the most abundant message (Figure S1A). We additionally analyzed these cells for basal Wnt/β-catenin signaling but did not see consistent or statistically significant activation of TOPFlash over the negative control FOPFlash reporter in any of the cell lines tested (data not shown). Although some of these cell lines have been reported to have endogenous Wnt/β-catenin activation due to upregulation of Wnts and silencing of secreted Wnt inhibitors [24], [25], [26], under our culture conditions there was no indication of a Wnt autocrine loop.

Because Wnt signaling can activate both β-catenin dependent and β-catenin-independent pathways, we tested if PORCN knockdown affected the proliferation and survival of these human breast cancer cell lines even in the absence of detectable endogenous β-catenin signaling. PORCN siRNAs siP7 and siP8 were able to produce at least 75% knockdown of PORCN mRNA in MDA-MB-231 cells (Figure 1G). Unexpectedly, this caused a marked decrease in cell proliferation over time (Figure 1D). These findings were extended in five additional breast cancer cell lines; RNAi-mediated knockdown of PORCN caused a statistically significant decrease in proliferation (Figure 1E) in each cell line tested. RNAi-mediated knockdown of PORCN has been reported to cause apoptosis in human lung cancer cells [27], although this finding could not be independently reproduced [28]. While we found a decrease in growth in a number of breast cancer cell lines, PORCN knockdown did not result in apoptosis or a shift in cell cycle distribution in any of the cancer lines tested (Figure S1B, and data not shown). Notably, PORCN knockdown does not affect the proliferation of all cells. For example, mesoderm-derived cancer lines such as HT-1080 fibrosarcoma cells and transformed BJ fibroblasts were not measurably affected (data not shown). Also, PORCN is not essential for the growth of embryonic stem cells and mouse embryo fibroblasts [12], [13]. Importantly, the growth effect of PORCN knockdown is transformation specific in epithelial cells. Transformed hMECs are sensitive to PORCN knockdown whereas immortalized HMECs are not affected (Figure 1F). Consistent with the finding that the effect of PORCN knockdown on growth is not dependent on Wnt signaling, the transformed hMECs do not display any evidence of Wnt/β-catenin autocrine signaling (data not shown).

The consequences of PORCN knockdown were examined further by assaying endogenous Wnt target genes in SK-BR-3 and MDA-MB-231 cells, as they had the largest proliferation defect after PORCN knockdown. These lines have been reported to have autocrine Wnt signaling [24], [29], although we could not detect this in TOPFLASH assays in our specific cell lines. PORCN knockdown in MDA-MB-231 cells led to a modest but statistically significant decrease in the abundance of transcripts encoding the Wnt target genes AXIN2, Cyclin D, and LEF1 (Figure 1G, top panel). A significant decrease of both AXIN2 and LEF1 mRNA was found in SK-BR-3 cells (Figure 1G, bottom panel). Taken together, PORCN knockdown decreased the growth rate of a number of transformed cell lines, and this correlated imperfectly with knockdown of Wnt/β-catenin target gene including AXIN2, Cyclin D and CMYC.

### Knockdown of PORCN slows tumor growth in an orthotopic mouse model

To address whether PORCN knockdown might slow the growth of tumor cells in a more complex environment where additional stroma-derived signals can also stimulate proliferation, we investigated the rate of establishment of tumors *in vivo*. MDA-MB-231 cells transfected with siControl or siP7 siRNA were orthotopically injected into NOD-SCID mice and tumor take was monitored. MDA-MB-231 cells with transient PORCN knockdown displayed a 2-week delay in tumor take compared to controls (Figure S1C). Delay in tumor formation suggested that PORCN gene function is important to tumor take and/or progression and encouraged us to pursue a stable knockdown approach.

**Figure 2:**
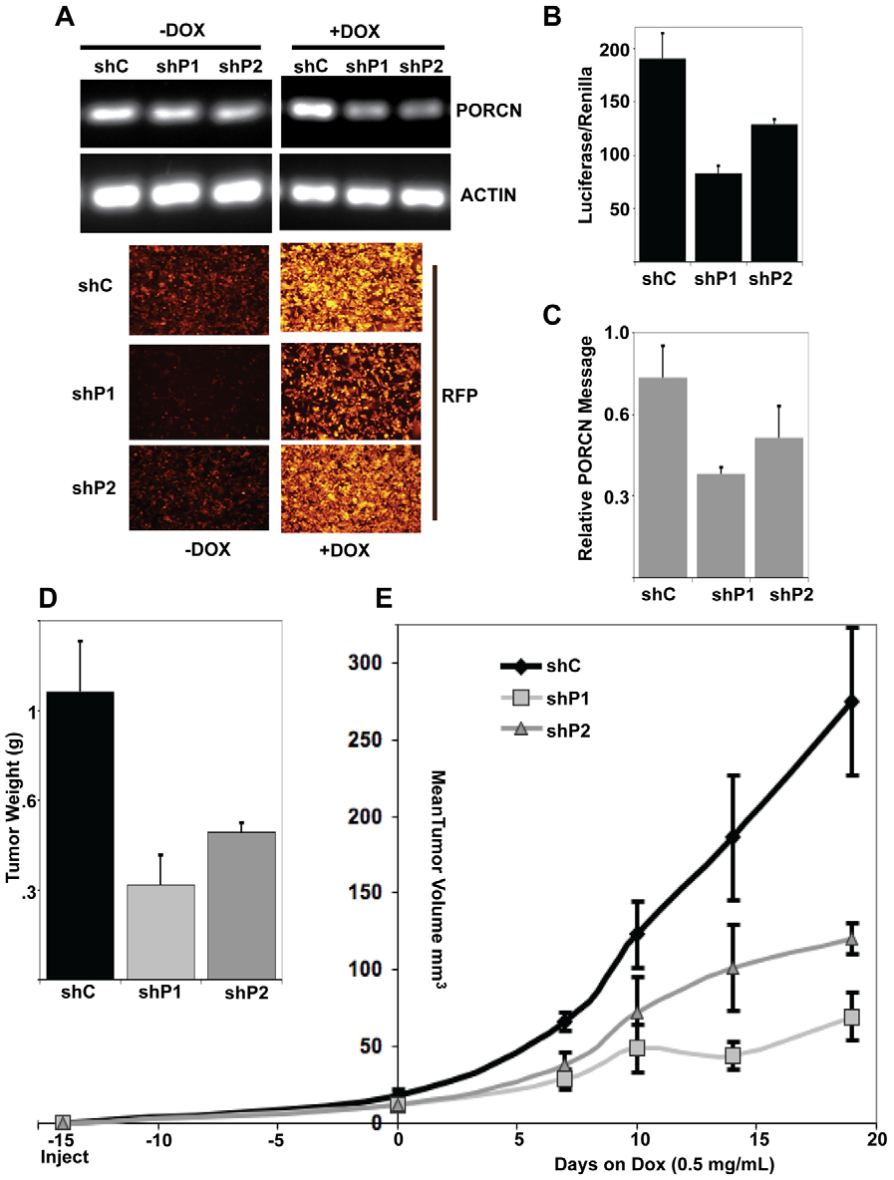
Inducible knockdown of PORCN delays cancer growth in an orthotopic xenograft model. A. Establishment of PORCN inducible knockdown. MDA-MB-231 cells with stable integration of pTRIPZ- shC (control), -shP1, or -shP2 shRNAmir were treated with 5 ng/mL Doxycycline (right) or vehicle control (left). PORCN and Actin mRNA were assessed by RT-PCR, and RFP expression was assessed by fluorescence microscopy. B. Inducible knockdown of PORCN inhibits Wnt/β-catenin signaling. The stable MDA-MB-231 cell lines were transiently transfected with WNT3A expression vector and SuperTOPflash and Renilla luciferase reporter plasmids, and then treated with 5 ng/mL Dox for 48 hrs before assessment of Wnt/β-catenin signaling as described. Histogram represents SuperTOPflash signaling relative to Renilla luciferase expression. C. Inducible knockdown of PORCN mRNA in orthotopic xenografts. The stable cell lines were injected orthotopically into the 4^th^ mammary fat pad of BALB/c nude mice. After establishment of palpable tumors (15 days), Dox was added to the water for a 7 day period, tumors were harvested and mRNA abundance was assessed by qRT-PCR. Histogram represents relative PORCN mRNA normalized to Actin mRNA. D. PORCN knockdown slows cancer growth. Tumors were harvested and weighed 19 days after the start of doxycycline treatment. E. Cancer growth is slowed by inducible PORCN knockdown. Tumor volume was measured by calipers at the indicated times.

To test if loss of PORCN would affect the growth of established tumors, we screened 25 mIR-30 based constructs and identified two independent microRNAs that provided robust PORCN knockdown. These were cloned into an inducible lentiviral vector, pTRIPZ, that provides dual expression of Red Fluorescent Protein (RFP) and the shRNAmir in the presence of doxycycline. MDA-MB-231 cells were generated with stably integrated pTRIPZ driving either scrambled shRNAmir (shControl) or one of two independent shRNAmirs against all splice variants of PORCN (here called shP1 and shP2). In cultured cells, addition of doxycycline led to induction of the shRNAmir with ∼75% reduction of PORCN message and high expression of RFP (Figure 2A). As expected, this decrease in PORCN mRNA in the MDA-MB-231 cells led to a decrease in WNT3A-activated signaling from the STF reporter (Figure 2B). To test whether PORCN knockdown could be induced *in vivo*, cells were injected orthotopically into BALB/c nude mice. When tumors reached a palpable size (∼0.2 cm), doxycycline was added to the drinking water. After 7 days on doxycycline, tumors were collected and analyzed for PORCN message. Both shP1 and shP2 tumors had a reduction in PORCN message, indicating that doxycycline is reaching the cells and inducing PORCN knockdown *in vivo* (Figure 2C).

To test whether PORCN knockdown affects tumor growth, the same cell lines were injected orthotopically into BALB/c nude mice. Tumors were allowed to reach ∼0.2 cm in diameter (about 2 weeks post injection), and then PORCN knockdown was induced by adding doxycycline to the water. After induction by doxycyline, there was a significant reduction in the rate of growth of tumors expressing either shP1 or shP2 (Figure 2E). This appeared to be dose dependent, as shP1 produced both a greater knockdown of PORCN and a greater reduction in tumor growth (Figure 2C and E). Once the control tumors reached ∼1 cm in diameter, the mice were sacrificed. Tumors were excised, weighed and measured. Tumors were significantly smaller and lighter if they contained the induced shP1 or shP2 shRNAmir (Figure 2D). These results demonstrate that knockdown of PORCN mRNA in an established orthotopic breast cancer results in a significant delay in tumor growth. Thus, PORCN plays a critical role in MDA-MB-231 cell proliferation both in culture, and in xenografts.

### Loss of PORCN affects cancer cell growth in a Wnt-independent manner

The above results show that knockdown of PORCN regulates proliferation of multiple breast cancer cell lines. The effect on proliferation is not due to off-target effects of the RNAi, since we obtained identical results with two independent siRNAs and two additional independent shRNAmirs against a total of 4 different regions of PORCN. Importantly, all the RNAi constructs were within coding sequence contained within each of the four human PORCN splice variants. Because the effect of PORCN knockdown did not correlate strongly with its effect on β-catenin dependent genes such as AXIN2, CYCLIN D, and CMYC, we considered the possibility that PORCN knockdown might decrease cell growth through a β-catenin independent autocrine Wnt loop or, alternatively, that PORCN has some additional, Wnt-independent functions. To differentiate between these possibilities, we inhibited Wnt secretion by two additional independent approaches.

**Figure 3:**
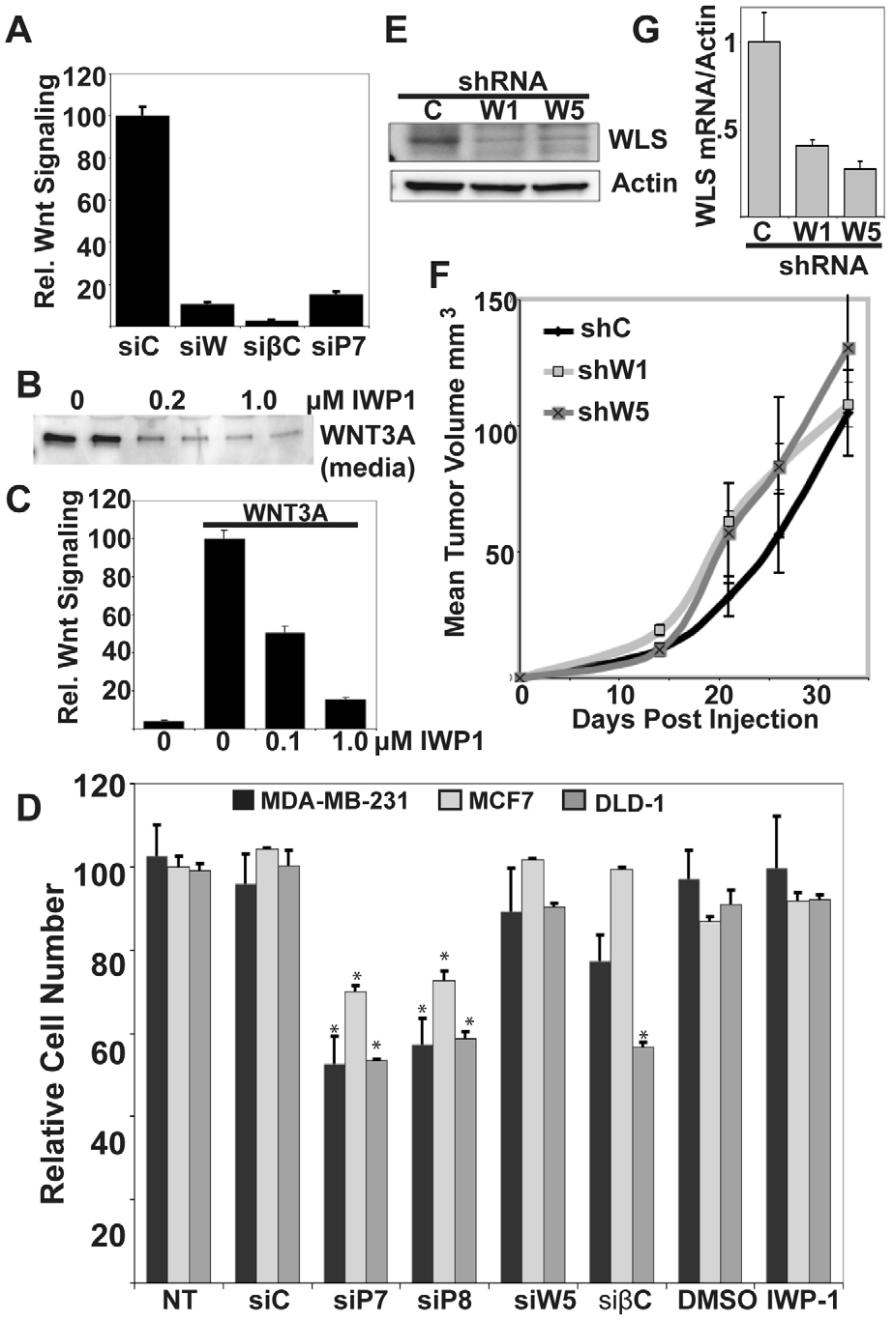
PORCN knockdown has Wnt/β-catenin-independent effects. A. WLS, β-catenin, and PORCN knockdown all inhibit Wnt/β-catenin signaling in STF3A cells. The following siRNAs were used at 100 nM: for PORCN, siP7; for WLS, siW5; for β-catenin, siβC11. B. IWP-1 inhibits WNT3A secretion. Conditioned medium from STF3A cells was assessed for WNT3A as described after cells were incubated for 16 hours in the presence of the indicated concentration of IWP-1. C. IWP-1 inhibits WNT3A-driven signaling in MDA-MB-231 cells. MDA-MB-231 cells were co-transfected with WNT3A and STF, and treated overnight with vehicle (DMSO) or indicated doses of IWP-1. D. The indicated cell lines were either transfected with siRNAs as above, or treated with IWP-1 or vehicle control. Total cell count at day 6 was assessed as described and compared with untreated cells. IWP-1 was used at 2 µM and refreshed every 24 h. E. Western blot of WLS in MDA-MB-231 cells stably expressing shC, shW1, and shW5 shRNAs. F. WLS knockdown does not slow tumor growth. MDA-MB-231 cells stably expressing shC, shW1, and shW5 shRNAs were injected orthotopically into BALB/C nude mice and tumor growth monitored as described. G. WLS message levels in tumors extracted from (E), assessed by qRT-PCR and normalized to Actin.

Acylation of Wnts by PORCN is required for their binding to the transport protein, WLS, which carries Wnts from the ER or Golgi to the plasma membrane prior to secretion [16]. As such, pharmacological inhibition of acylation and loss of WLS both inhibit Wnt secretion akin to PORCN knockdown [17], [18]. Insofar as we know to date, all mammalian Wnts require both PORCN and WLS for secretion. As expected, siRNA knockdown of WLS and PORCN all similarly and effectively inhibited WNT3A-driven signaling (Figure 3A). PORCN enzymatic function can be inhibited with the small molecule inhibitor IWP-1 [14]. Similar to published results, we found that IWP-1 inhibits Wnt secretion into the medium with an IC_50_ of ∼200 nM (Figure 3B). IWP-1 also effectively inhibits WNT3A-driven signaling in MDA-MB-231 cells at similar doses (Figure 3C). Using these alternative approaches, we examined the consequence of decreased Wnt secretion on cancer cell growth.

Surprisingly, unlike PORCN knockdown, knockdown of WLS had little or no effect on the proliferation of any cancer cell line tested, including MDA-MB-231, MCF7, DLD-1, SK-BR-3, T47D, and HeLa cells (Figure 3D and data not shown). Likewise, β-catenin knockdown did not affect cell growth in MDA-MB-231 or MCF7 cells. Confirming the efficacy of knockdown, loss of β-catenin significantly decreased the proliferation of DLD-1 cells, which have stabilized β-catenin due to a mutation in the adenomatous polyposis coli (APC) gene (Figure 3D). The small effect of β-catenin knockdown on MDA-MB-231 cell proliferation in this experiment was not seen in repeat experiments. Furthermore, addition of IWP-1 at doses that give >80% reduction of Wnt/β-catenin signaling had no effect on the cell growth or viability of MDA-MB-231, MCF7, and DLD-1 cells, lines that were sensitive to both PORCN siRNA and shRNA (Figure 3D). We extended this result to a panel of other cancer cell lines and also found no effects of IWP-1 or the related molecule IWP-2 on cell growth and viability (Figure S2). These results demonstrate that blockade of Wnt secretion by WLS knockdown or IWP treatment did not alter cancer cell proliferation and survival, implying that these cells do not depend on autocrine Wnt signaling. Importantly, this data suggests that loss of PORCN may therefore affect growth in a Wnt independent manner.

### Loss of WLS does not affect tumor take or growth

Because of the surprising lack of effect of WLS knockdown in cell culture, we sought to extend this result to a tumor model. MDA-MB-231 cells were stably transduced with one of two shRNA targeting WLS (shW1 and shW5) or a control shRNA, (shC). Both shRNA targeting WLS gave an effective knockdown at the mRNA (>80%) and at the protein level (Figure 3E). These cells were injected orthotopically into BALB/c nude mice and tumor progression was monitored. Tumors from all three lines grew at a very similar rate (Figure 3F). When tumors reached ∼1 cm in diameter, they were removed and assessed for persistence of WLS knockdown. Both shW1 and shW5 maintained a >60% knockdown of WLS in the tumors (Figure 3G). Thus, while WLS knockdown is effective, persistent, and diminishes β-catenin signaling, it has no effect on MDA-MB-231 cell growth or xenograft tumors. Taken together with the effect of PORCN knockdown on tumor growth (Figure 2E), this data further supports the idea of a Wnt-independent role of PORCN in cancer cell growth *in vivo*

### Wild Type and mutant PORCN both rescue growth effects

**Figure 4:**
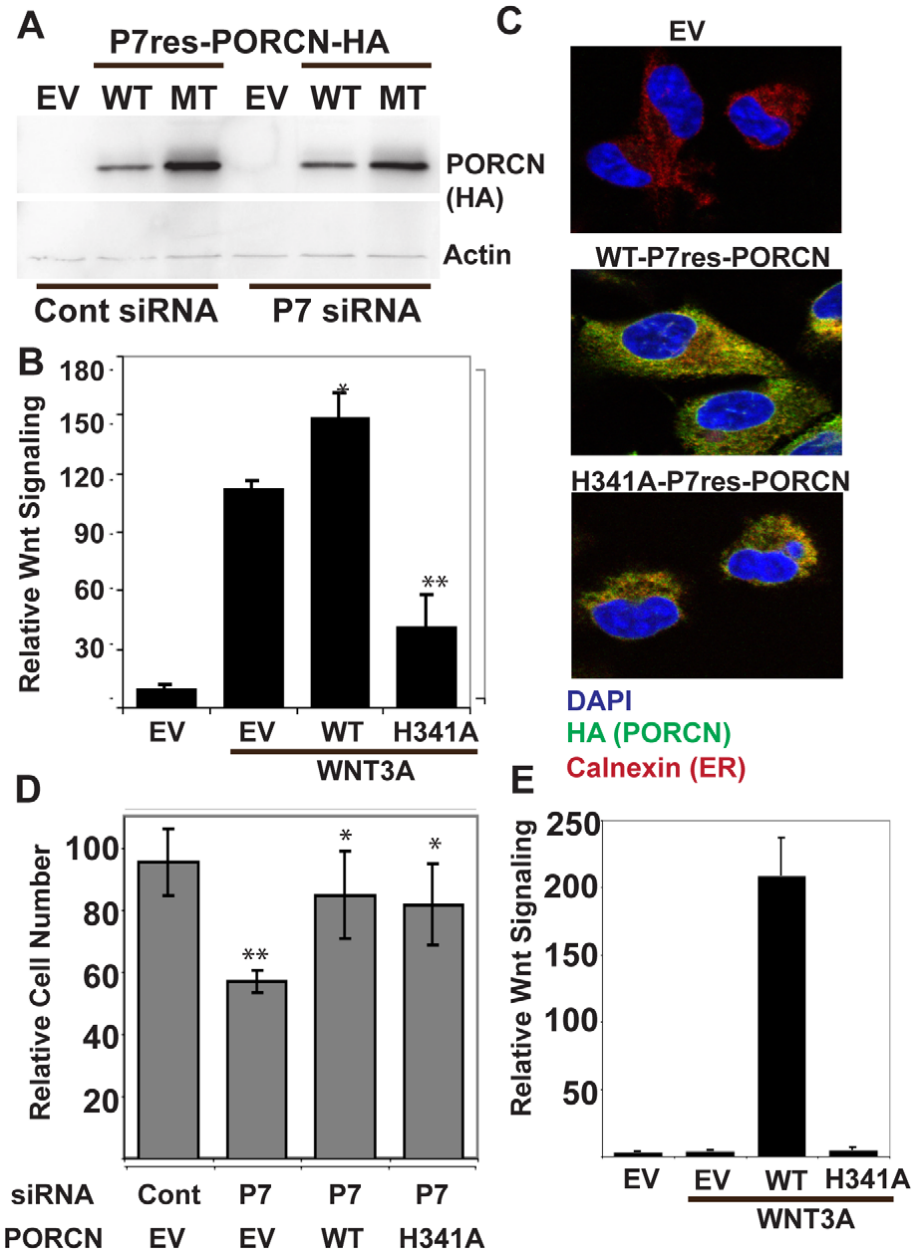
Wild type and mutant PORCN both rescue the slow growth phenotype. A. Western blot analysis of MDA-MB-231 cells transfected with P7 immune constructs of HA-tagged WT and H341A PORCN. The PORCN construct contains 2 additional silent mutations that render it immune to P7 siRNA knockdown. EV, empty vector; WT, wildtype PORCN-HA; MT, H341A-PORCN-HA. B. Wildtype but not mutant PORCN stimulates Wnt/β-catenin signaling. MDA-MB-231 cells were co-transfected with plasmids encoding for WT- and H341A-PORCN, WNT3A, and the SuperTOPflash reporter. H341A-PORCN has a significant dominant negative effect on Wnt/β-catenin signaling. C. Wildtype and mutant PORCN are appropriately localized. Indirect immunofluorescence microscopy was performed with anti-HA antibody and the endoplasmic reticulum marker calnexin. D. Wildtype and dominant negative H341A PORCN are equally effective at rescuing the growth defect caused by PORCN knockdown. MDA-MB-231 were co-transfected with P7 siRNA and PORCN expression constructs as indicated. Cell numbers were measured as described. Histogram represents cell number at day 5. *, p<0.01 for ability of constructs to rescue slowed proliferation; **, p<0.01 for ability of PORCN knockdown to slow proliferation relative to control. E. WT but not H341A-PORCN is able to rescue Wnt/β-catenin signaling. PORCN null ES cells were co-transfected with plasmids encoding WT- and H341A-PORCN, WNT3A, and the SuperTOPflash reporter. In the absence of ectopic PORCN in these ES cells, there is no WNT3A-driven signaling.

PORCN knockdown slowed cancer proliferation, unlike inhibition of PORCN enzymatic activity with the small molecule IWP-1. This paradox suggested that PORCN protein might play a required structural role in cells. To confirm that loss of PORCN protein is responsible for the observed growth effects, we sought to rescue this phenotype by using an siRNA-immune version of mouse PORCN-D. Using an MSCV-3XHA-tagged mPORCN-D construct [4], we used site directed mutagenesis to give immunity to siP7 siRNA. To make catalytically inactive PORCN, we mutated H341, an invariant MBOAT catalytic residue predicted to participate in PORCN catalytic activity. Experiments in several PORCN-null cell lines confirmed that mPORCN(H341A) is completely catalytically inactive (Figure 4E and data not shown). In addition, PORCN(H341L) has recently been shown to be catalytically inactive in PORCN null ES cells [13]. In transient transfections, the PORCN mutant protein was expressed at levels similar to wildtype PORCN and acts in a dominant negative manner, effectively inhibiting both Wnt secretion and TCF-driven luciferase expression in STF3A cells (Figure S3, A–C). Using siRNA-immune versions of these constructs, we cotransfected MDA-MB-231 cells with siP7 siRNA and showed it had no effect on abundance of the ectopic PORCN protein (Figure 4A, top panel), confirming immunity to the siP7 siRNA. MDA-MB-231 cells expressing wildtype mPORCN have increased WNT3A-driven STF activity (Figure 4B). Notably, MDA-MB-231 cells expressing H341A mPORCN have reduced WNT3A-driven β-catenin signaling activity, consistent with a dominant negative function in MDA-MB-231 cells as well. Both wildtype and H341A PORCN co-localize with calnexin (Figure 4C), an ER marker [30], consistent with previous reports showing PORCN localization to the ER [4].

To test if the growth defect caused by PORCN knockdown could be rescued by re-expression of a catalytically inactive, dominant negative PORCN, growth assays were performed in MDA-MB-231 cells transiently transfected with either empty vector (EV), wildtype (WT), or H341A-PORCN expression constructs and co-transfected with siControl or siP7. As seen previously, the MDA-MB-231 cells responded to PORCN knockdown with a reduction in cell growth (Figure 4D). Ectopic WT-PORCN significantly rescued the growth when endogenous PORCN was knocked down (Figure 4D). Remarkably, cells exhibited identical growth rescue when catalytically inactive H341A-PORCN was expressed (Figure 4D). Rescue with catalytically inactive H341A-PORCN is consistent with our finding that pharmacological inhibition of PORCN and knockdown of WLS have no effect on MDA-MB-231 cell proliferation. Rescue of the proliferation defects by a catalytically inactive mutant suggests that PORCN is playing an essential, possibly structural, role in the endoplasmic reticulum that is independent of its ability to support Wnt acylation and secretion.

### PORCN Regulates a Distinct Subset of Genes in a Wnt Independent Manner

**Figure 5:**
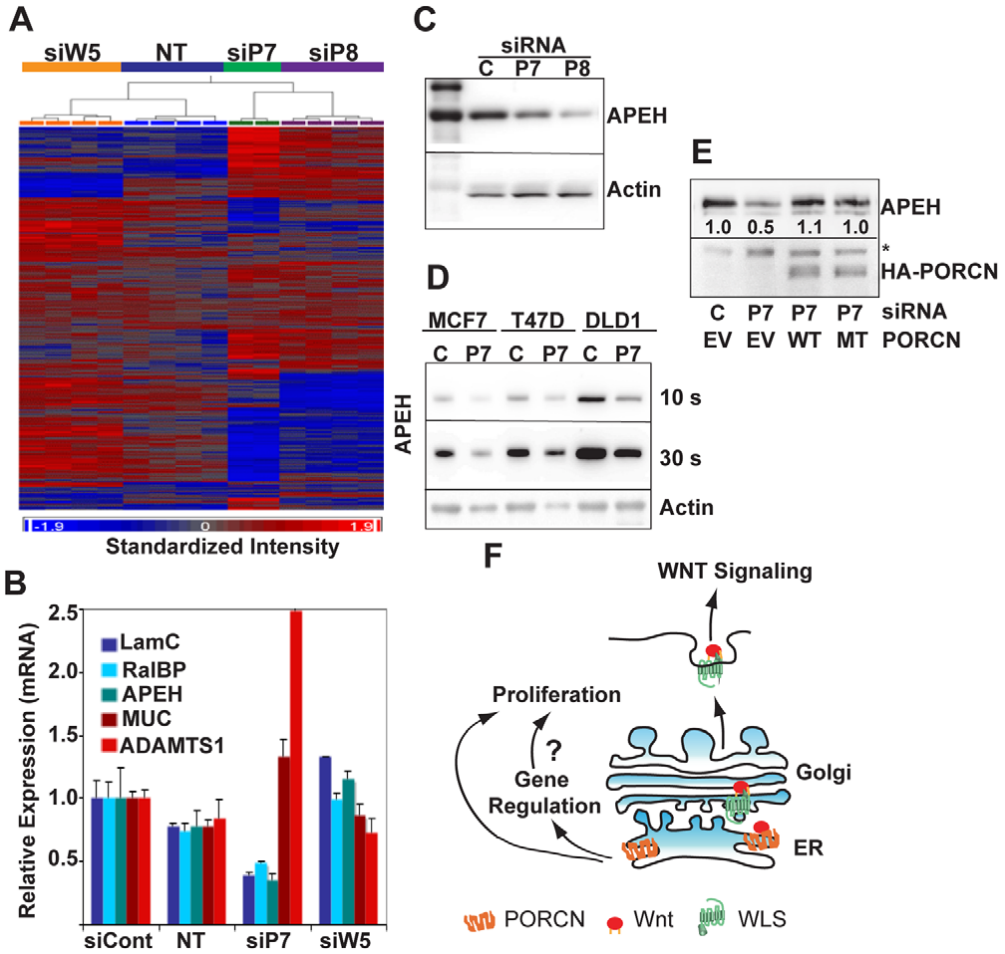
PORCN regulates a subset of genes in a Wnt-independent manner. A. Gene expression analysis of MDA-MB-231 cells 48 hours after mock transfected (NT) or transfected with 100 nM of indicated siRNA for 48 hrs. PORCN and WLS differentially regulate a distinct subset of genes. B. To illustrate genes differentially regulated by PORCN, the abundance of the indicated transcripts were assessed by qRT-PCR 48 hours after transfection of MDA-MB-231 cells with the indicated siRNAs C. Two independent PORCN siRNAs cause decrease in APEH protein. Immunoblot analysis of APEH and Actin from MDA-MB-231 cells treated with 100 nM Control (C), PORCN7 (P7) or PORCN8 (P8) siRNA for 72 hrs. D. PORCN knockdown triggers loss of APEH in multiple cell lines. Immunoblot analysis of APEH from MCF7, T47D, and DLD-1 cells transfected with 100 nM Control (C), or PORCN7 (P7) siRNAs for 72 hrs. Top panel is 10 s exposure, middle panel is 30 s exposure. Actin (bottom panel) serves as a load control. E. Loss of APEH is blocked by RNAi-immune wildtype and mutant PORCN. Immunoblot analysis of APEH and HA-tagged PORCN in MDA-MB-231 cells co-transfected with P7-resistant WT or H341A PORCN and 100 nM Control (C) or PORCN7 (P7) siRNA for 48 hrs. * = non-specific band recognized by anti-HA antibody, used as load control. Experiment done in duplicate with same results. F. Model of PORCN's function in transformed epithelial cells. PORCN is involved in the acylation and secretion of Wnt proteins. Independent of its role in Wnt secretion, PORCN regulates proliferation and gene expression. The role in gene expression may lead to changes in proliferation.

Because of the surprising differences between loss of PORCN and WLS on cell growth, we further investigated whether PORCN and WLS function in distinct pathways by examining the effect of knockdown on gene expression profiles. MDA-MB-231 cells were transfected with mock, siControl, siP7, siP8, or siW5 siRNA. mRNA was harvested 48 hrs later for gene expression analysis. Gene expression changes following WLS knockdown did not differ much from control siRNA, consistent with the low levels of autocrine signaling in our cell lines. Unexpectedly, the expression profiles after PORCN or WLS knockdown were significantly different, with only a few genes coordinately regulated (Figure 5A). Interestingly, distinct subsets of genes were up- or down-regulated by PORCN knockdown that were unchanged after WLS knockdown, a finding that was repeated and confirmed by qPCR (Figure 5B). For example, PORCN knockdown produced a 60% reduction in LAMC expression and a 2.5-fold increase in ADAMTS1 expression, while 90% knockdown of WLS had no effect on these genes. One of the genes whose expression was most significantly down-regulated by PORCN knockdown was N-acylaminoacyl-peptide hydrolase (APEH). APEH catalyzes the hydrolysis of N-acetylated amino acids from small acetylated peptides or proteins [31]. We confirmed that PORCN knockdown caused downregulation of both APEH mRNA (Figure 5B) and protein (Figure 5C) in MDA-MB-231 cells. The regulation of APEH by PORCN was consistent across cell lines; for example, MCF7, T47D, and DLD-1 cells also exhibited APEH downregulation with PORCN knockdown (Figure 5D). The regulation of APEH is Wnt-independent, as WLS knockdown has no effect on APEH mRNA (Figure 5B) or protein levels (data not shown). IWP-1 treatment also had no effect on APEH levels (data not shown). Importantly, APEH expression in PORCN-knockdown cells was rescued by ectopic expression of both wildtype and H341A-PORCN (Figure 5E), further demonstrating that catalytically inactive PORCN regulates pathways independent of its function of palmitoylating Wnt proteins.

The transcription of ADAMTS1, APEH, LAMC, and RALBP1 has no obvious functional relationship with PORCN, which is an ER-resident protein. The effects on transcription could be a direct consequence of disruption of an ER function leading to changes in intracellular signaling, or an indirect and secondary consequence of loss of PORCN function. We could find no evidence of activation of the ER stress response pathway (data not shown). We also tested whether direct knockdown of APEH, RALBP1, or LAMC in MDA-MB-231 cells recapitulated the PORCN knockdown slow growth phenotype. However, no single gene knockdown reproduced this effect. We speculate that a combination of genes that are up- or down-regulated by PORCN knockdown are responsible for the altered proliferation seen in transformed epithelial cells. Taken together, the data indicate PORCN plays two roles in the ER, one to acylate Wnts, and independently, one to participate non-enzymatically in a pathway that is rate-limiting for transformed epithelial cell growth (Figure 5F).

## Discussion

In this report, we investigated the role of PORCN in proliferation and survival in tumorigenesis. We found that PORCN knockdown inhibits the growth of a number of epithelial cancer cell lines. Surprisingly, these cells have little or no evidence of autocrine Wnt signaling, suggesting that the effect on proliferation is independent of Wnt/β-catenin signaling. Supporting this, unlike PORCN knockdown, WLS knockdown, β-catenin knockdown, and pharmacological inhibition of PORCN do not affect growth of these cell lines. This unexpected function of PORCN in cell growth is also independent of its catalytic function as an acyltransferase, as we can rescue both growth and changes in gene expression with a catalytically inactive, dominant negative mutant of PORCN. Taken together, our data strongly suggests that PORCN has a rate-limiting, Wnt-independent function in epithelial cancer cells.

There is increasing recognition that enzymes can have additional functions alongside their catalytic roles. This phenomenon is called ‘moonlighting’, where proteins perform multiple different functions using the same protein domain (reviewed recently by [32], [33]. Moonlighting appears to be more common in highly conserved enzymes, although the known proteins that can moonlight are rapidly expanding. Some examples of moonlighting include metabolic enzymes such as glyceraldehyde-3-phosphate dehydrogenase and lactate dehydrogenase and signaling kinases such as Akt, Erk2 and the pyruvate kinase splice variant M2 [34]. Our data suggest that the evolutionarily ancient PORCN protein has also evolved additional roles that are independent of its catalytic function.

An intriguing insight into PORCN moonlighting function is that it is not rate limiting for growth in all cell types, but rather only in transformed epithelial cells. Knockdown of PORCN did not affect the proliferation of HEK 293 cells, HT1080 fibrosarcoma cells, non-transformed HMECs, or BJ foreskin fibroblast cell lines regardless of transformation status. Complete genetic knockout of PORCN also did not affect the proliferation of pluripotent stem cells, as murine ES cells with inactivation of PORCN are able to both grow in culture [12], [13], [35] and form teratomas in nude mice (TMC, manuscript in preparation). It is known that cells undergo significant stresses during malignant transformation, including changes in metabolic flux, oxidative stress, checkpoint activation and apoptotic signaling. It is possible that PORCN's moonlighting role becomes rate limiting as part of the adaptation to the oncogenic transformation in certain epithelial cancers. These epithelial-specific functions may provide insight into the mechanism of PORCN's moonlighting function.

The exact moonlighting function of PORCN remains unknown. A recent paper investigating PORCN in Non Small Cell Lung Carcinoma illustrated that loss of PORCN downregulates S100P at the mRNA and protein levels in a β-catenin independent manner [28], similar to what we have shown for APEH. As PORCN is an integral ER membrane protein, it seems most likely that its loss disrupts an ER process. Further insights may come from identifying PORCN-interacting proteins in the ER, and/or by following the signaling pathways that alter expression of APEH.

Mutations in PORCN cause an X-linked inherited disorder called Focal Dermal Hypoplasia (FDH) [36], [37]. While FDH is thought to be the result of defective Wnt signaling, our finding raises the possibility that the disruption of the moonlighting function of PORCN may in some cases contribute to the pathogenesis of the disease. In FDH, there are over 25 different mutations of PORCN found that have not been extensively characterized biochemically, and the severity of the disease varies greatly [38]. It is possible that a subset of these mutations might alter proliferation in a developmental context in a Wnt-independent manner, especially since many types of mutations lead to premature stop codons. Evidence against this is our data showing the moonlighting function is not obviously rate limiting in fibroblasts and non-transformed epithelial cells in culture. However, PORCN expression has been identified in tissues not associated with Wnt ligand expression during the development of mice, such as the anterior visceral endoderm [12]. Our data raises the possibility that PORCN expression in these tissues may be important for Wnt-independent functions.

The goal of this study was to investigate the role of PORCN in tumorigenesis. We have discovered that PORCN knockdown affects cancer cell growth and tumorigenesis in a Wnt-independent manner. This study is the first to implicate a Wnt-independent role of PORCN in cancer cells. Further investigation of the Wnt-independent roles of PORCN in the endoplasmic reticulum may provide insights into both novel functions of PORCN and the endoplasmic reticulum in proliferation.

## Supporting Information

Figure S1 **Analysis of PORCN and PORCN knockdown in breast cancer cells and tumorigenesis.** A. PORCN message relative to Actin in a panel of breast cancer cell lines B. Tumor take of MDA-MB-231 cells transfected with 100 nM of siC or siP7 and injected orthotopically into nude mice. Transient knockdown of PORCN resulted in a 2 week delay in tumor take. C. FACS profile of MDA-MB-231 cells following 72 hr transfection with 100 nM of Cont, P7, P8, or W5 siRNAs. Although total cell number was less in the P7 and P8 treated cells, there is no significant induction in apoptosis or change in cell cycle profile.
(TIF)

Figure S2 **IWP-1 and IWP-2 small molecule inhibitors do not affect cell growth or viability.** A. Cell viability of a panel of breast cancer cells treated with IWP-2 for a 5 day period. *These concentrations of IWP-2 were not entirely soluble and formed visible precipitates. B. Growth assay of a panel of cancer cells treated with 1 µM IWP-1. Following 6 days of treatment, the cells were fixed with MeOH and stained with crystal violet.
(TIF)

Figure S3 **H341A-PORCN affects Wnt signaling and WNT3A secretion in STF cells.** A. Relative Wnt signaling in STF3A cells transfected with empty vector or WT or H341A mPORCN-D. B. WNT3A secretion into the media from STF3A cells transfected with empty vector or WT or H341A mPORCN-D. C. Relative expression of WT or H341A mPORCN-D transfected into STF3A cells.
(TIF)
