# Supplementary figures and images for: PORCN Moonlights in a Wnt-Independent Pathway That Regulates Cancer Cell Proliferation
Source: PLoS One. 2012 Apr 11;7(4):e34532. doi: 10.1371/journal.pone.0034532 (PMC3324524; doi:10.1371/journal.pone.0034532)

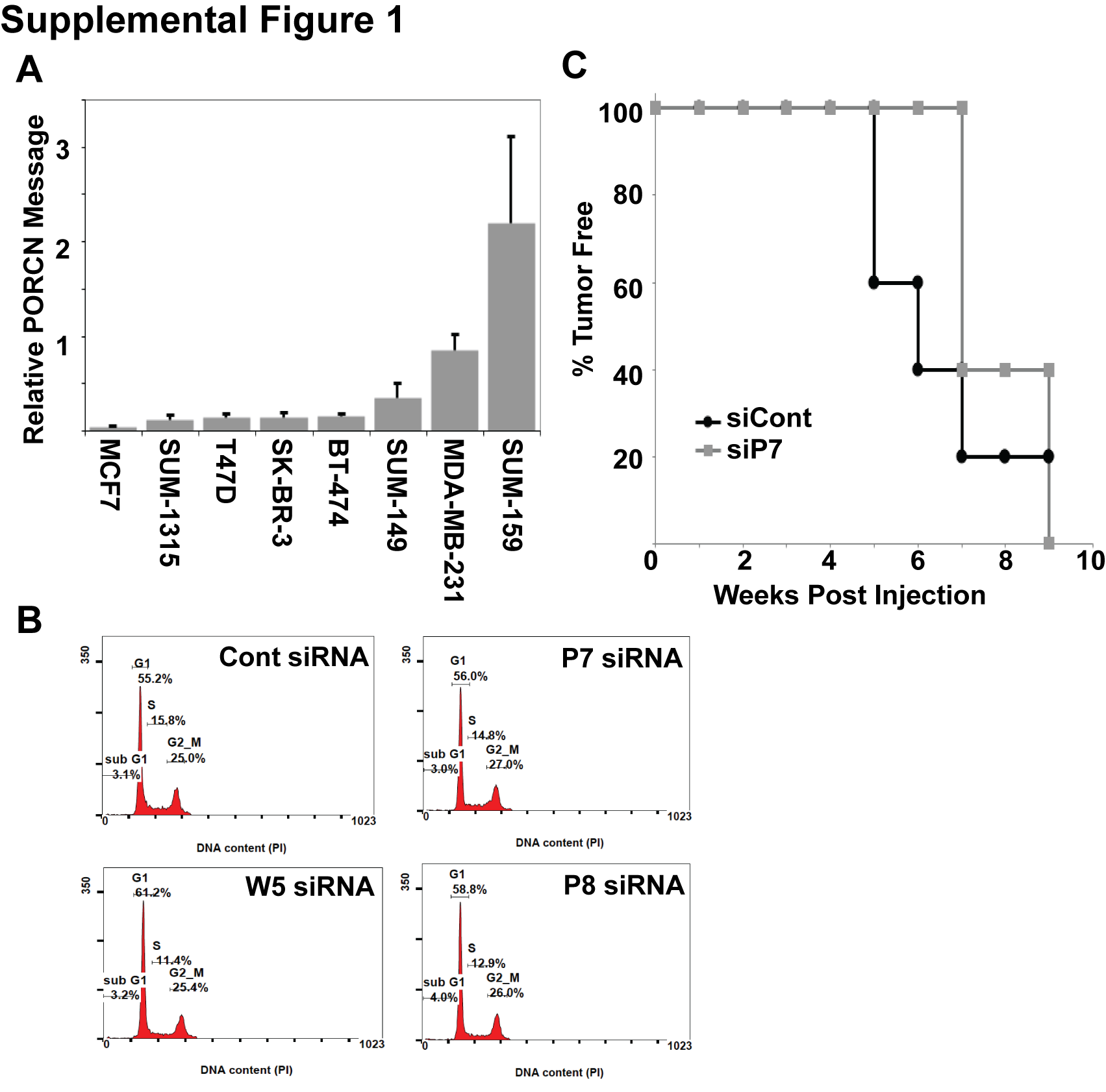

Supplement: Figure S1 — Analysis of PORCN and PORCN knockdown in breast cancer cells and tumorigenesis. A. PORCN message relative to Actin in a panel of breast cancer cell lines B. Tumor take of MDA-MB-231 cells transfected with 100 nM of siC or siP7 and injected orthotopically into nude mice. Transient knockdown of PORCN resulted in a 2 week delay in tumor take. C. FACS profile of MDA-MB-231 cells following 72 hr transfection with 100 nM of Cont, P7, P8, or W5 siRNAs. Although total cell number was less in the P7 and P8 treated cells, there is no significant induction in apoptosis or change in cell cycle profile. (TIF) [file pone.0034532.s001.tif]

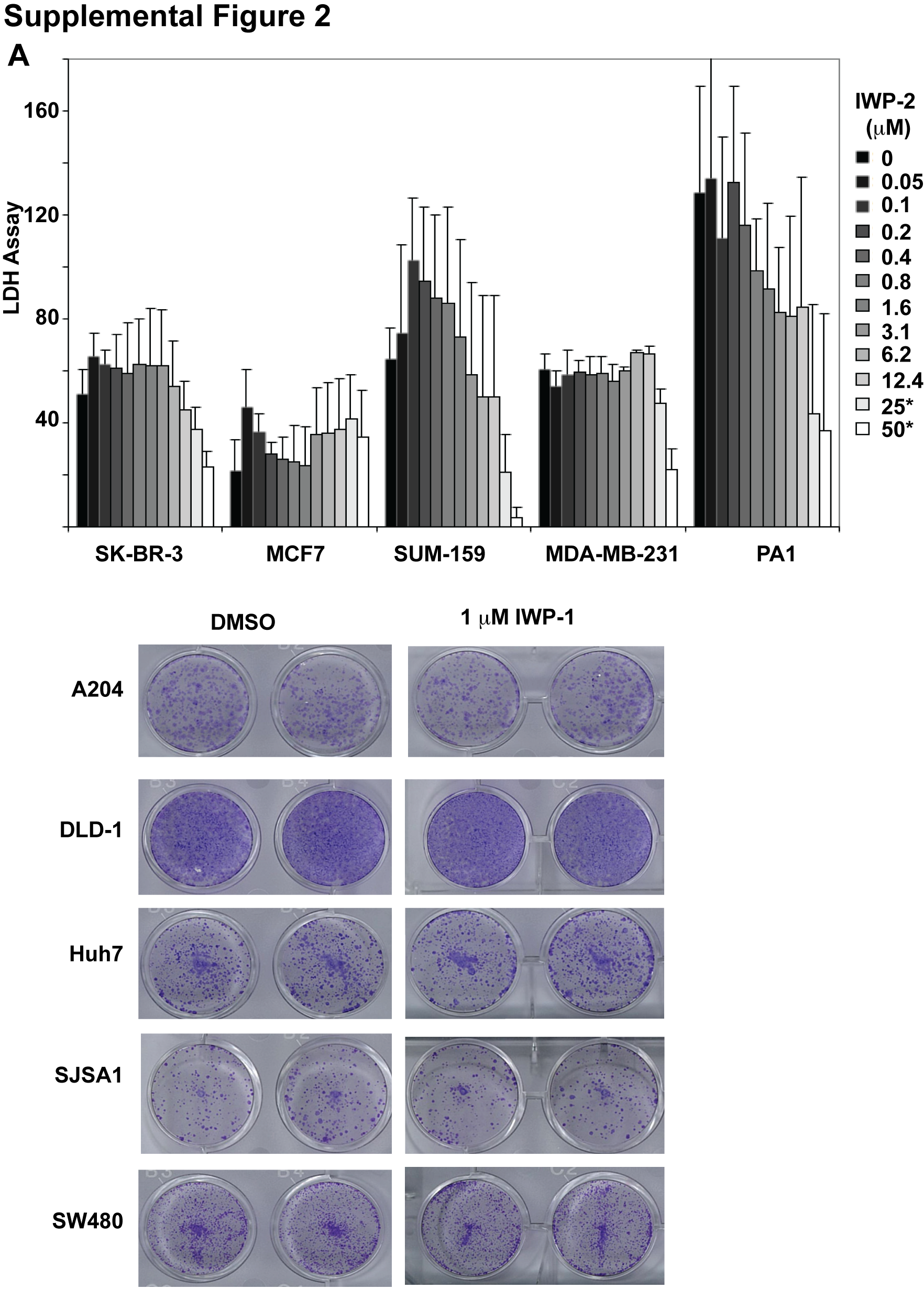

Supplement: Figure S2 — IWP-1 and IWP-2 small molecule inhibitors do not affect cell growth or viability. A. Cell viability of a panel of breast cancer cells treated with IWP-2 for a 5 day period. *These concentrations of IWP-2 were not entirely soluble and formed visible precipitates. B. Growth assay of a panel of cancer cells treated with 1 µM IWP-1. Following 6 days of treatment, the cells were fixed with MeOH and stained with crystal violet. (TIF) [file pone.0034532.s002.tif]

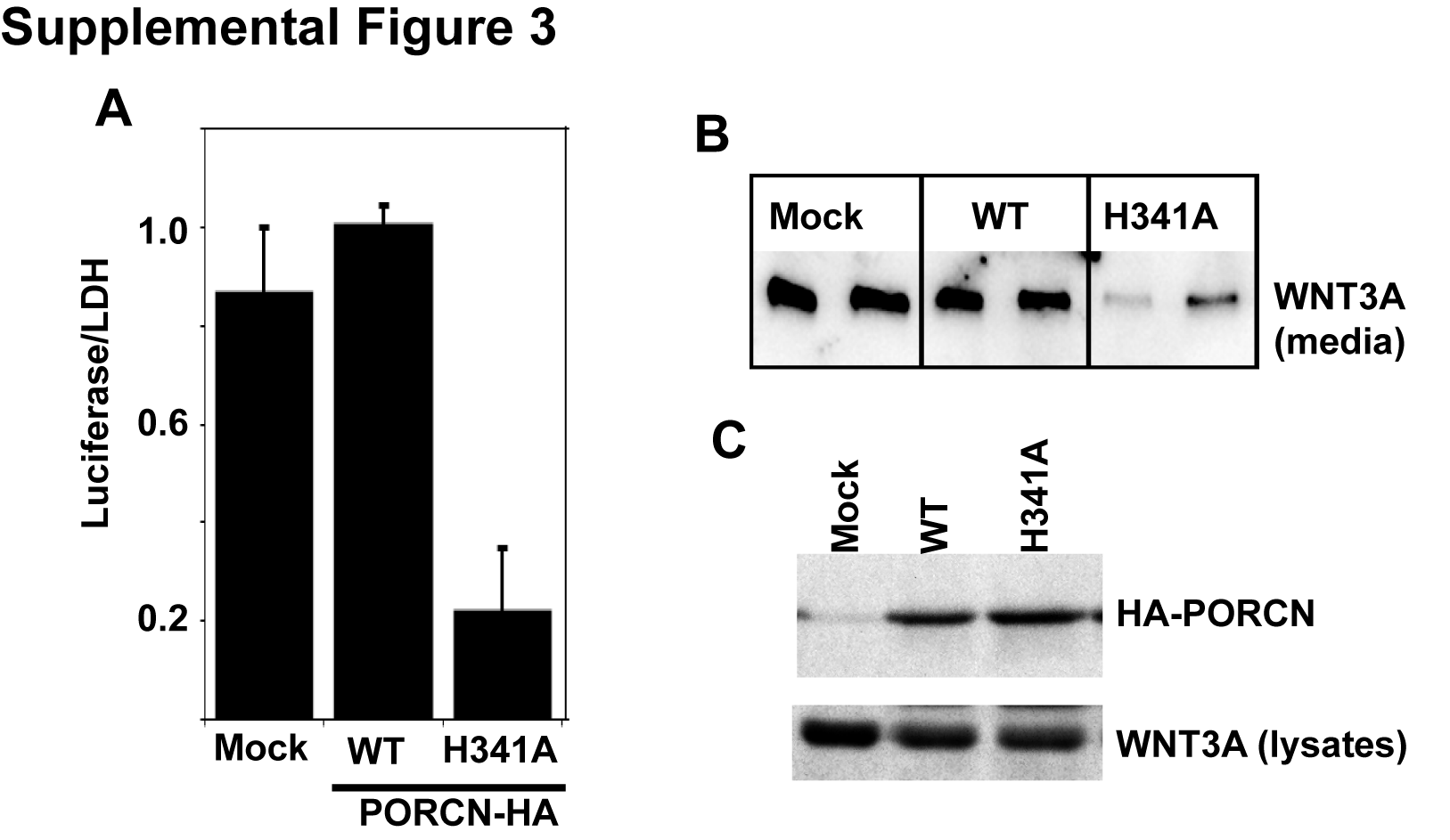

Supplement: Figure S3 — H341A-PORCN affects Wnt signaling and WNT3A secretion in STF cells. A. Relative Wnt signaling in STF3A cells transfected with empty vector or WT or H341A mPORCN-D. B. WNT3A secretion into the media from STF3A cells transfected with empty vector or WT or H341A mPORCN-D. C. Relative expression of WT or H341A mPORCN-D transfected into STF3A cells. (TIF) [file pone.0034532.s003.tif]
